# Heart failure and predictors of outcome in eosinophilic myocarditis: a real-world cohort study

**DOI:** 10.3389/fmed.2026.1887831

**Published:** 2026-08-26

**Authors:** Fabian T. H. Ullrich, Ulf Schönermarck, Katrin Milger, Antoine Murray, Matthias B. Thaler, Didzis Gailis, Julia Lichtnekert, Judith E. Spiro, Lorenz Schimang, Alla Skapenko, Ulrich Grabmaier, Hendrik Schulze-Koops

**Affiliations:** 1 Division of Rheumatology and Clinical Immunology, Department of Medicine IV, LMU University Hospital, LMU Medizin, LMU Munich, Munich, Germany; 2 Division of Nephrology, Department of Medicine IV, LMU University Hospital, LMU Medizin, LMU Munich, Munich, Germany; 3 Clinical Department of Pulmonology, Medical University of Graz, Graz, Austria; 4 Division of Respiratory Medicine, Department of Internal Medicine, Lung Research Cluster, Medical University of Graz, Graz, Austria; 5 Department of Medicine V, LMU University Hospital, LMU Medizin, LMU Munich, Munich, Germany; 6 Comprehensive Pneumology Center, German Center for Lung Research (DZL), Munich, Germany; 7 Practice of Rheumatology Soest, Soest, Germany; 8 Department of Radiology, LMU University Hospital, LMU Medizin, LMU Munich, Munich, Germany; 9 Department of Medicine I, LMU University Hospital, LMU Medizin, LMU Munich, Munich, Germany

**Keywords:** biomarkers, eosinophilic granulomatosis with polyangiitis, eosinophilic myocarditis, heart failure, hypereosinophilic syndrome, predictors of outcome, cardiac function

## Abstract

**Objective:**

In eosinophilic diseases, myocardial involvement critically impacts prognosis. Given the rarity and heterogeneity of eosinophilic myocarditis (EM), diagnosis and risk-adapted treatment remain challenging, and longitudinal data on cardiac function are scarce. Therefore, this study investigated the course of cardiac function and aimed to identify prognostic biomarkers in a real-world EM cohort across inflammatory disorders.

**Methods:**

Clinical, laboratory, and imaging data of all patients with eosinophilic myocarditis treated at Ludwig-Maximilians-University Hospital in Munich between January 2010 and May 2025 were reviewed. Descriptive and inferential statistics were applied to delineate disease courses, treatment regimens, and outcomes.

**Results:**

Of 419 patients screened, 51 had EM (51% female patients, mean age 50 ± 14 years). Underlying diseases included eosinophilic granulomatosis with polyangiitis (EGPA, *n* = 29), idiopathic hypereosinophilic syndrome (i-HES, *n* = 16), drug-associated/hypersensitivity myocarditis (*n* = 7; 4 with EGPA/i-HES), and idiopathic EM (*n* = 3). Mean left-ventricular ejection fraction (LVEF) was impaired at baseline (48 ± 17%) and after a median of 34 months [interquartile range 16–79] (*Δ* + 3 ± 12%, *p* = 0.57). Laboratory parameters improved (*p* < 0.001); however, LVEF recovered predominantly in patients with baseline evidence of active inflammation on cardiac magnetic resonance (CMR) or endomyocardial biopsy (EMB) (41 ± 15% to 48 ± 10%; *p* < 0.05). Median left-ventricular end-systolic/end-diastolic volume indices (LVESVI/-EDVI) increased over time (50 [28–65] to 56 [33–84] and 85 [68–98] to 97 [79–132] mL/m^2^, respectively; *p* < 0.05), suggesting cardiac remodeling. Glucocorticoids were broadly applied, whereas steroid-sparing drugs were used less frequently. Five patients died, all had reduced baseline LVEF. Age- and sex-adjusted baseline NT-proBNP (log_10_), LVEF, and the Sartorelli score, a published composite score (incorporating LV dysfunction, arrhythmias, CMR, and serum troponin), were significantly associated with follow-up LVEF, LVEDVI and LVESVI (rho/*r* = ±0.44–0.72, *R*^2^ = 0.19–0.64; *p* < 0.05–<0.0001).

**Conclusion:**

In this real-world cohort, EM severely impacted multiple domains of cardiac function. Treatment patterns were heterogeneous. Baseline NT-proBNP, LVEF, and the Sartorelli score were associated with long-term LVEF and LV remodeling. Baseline evidence of active myocardial inflammation on CMR and/or EMB was associated with subsequent LVEF improvement. Together, these findings support the utility of multimodal biomarkers for risk stratification in EM but require external validation before informing routine care. Prospective multicenter studies using standardized diagnostics and treatment regimens are needed to translate these findings into biomarker-guided management of eosinophilic cardiomyopathy.

## Introduction

Eosinophilic myocarditis (EM) is a rare inflammatory heart disease driven by infiltration of eosinophilic granulocytes into myocardial tissue. It may occur in the context of systemic inflammatory-rheumatic diseases (IRD) or without extracardiac involvement (1). Regardless of etiology, EM can cause severe myocardial injury and persistent cardiomyopathy. Eosinophilic granulomatosis with polyangiitis (EGPA), a systemic vasculitis of small- to medium-sized vessels, hypereosinophilic syndrome (HES), and (drug-)hypersensitivity reactions are the most common eosinophil-associated disorders (EAD) in which cardiac involvement constitutes a life-threatening manifestation. Whether phenotype and clinical course differ by underlying disorder remains unclear. Overall prognosis is poor, with clinical presentations ranging from asymptomatic to sudden cardiac death (1, 2).

Due to the rarity and heterogeneity of underlying EAD, studies addressing EM as a distinct entity are scarce, and standardized diagnostic and therapeutic approaches including validated biomarkers are lacking. In 2024, the International Eosinophil Society emphasized the need for biomarkers in rare EAD (3). Although endomyocardial biopsy (EMB) and cardiac magnetic resonance (CMR) are considered diagnostic gold standards (4), diagnosis of IRD-related EM in practice often relies on symptoms, laboratory findings, and cardiac dysfunction after exclusion of alternative causes (5, 6).

Immunosuppressive treatment is crucial in eosinophilic IRD; however, evidence for cardiac involvement remains limited (7, 8). Given the organ-threatening nature, high-dose glucocorticoids (GC) are frequently combined with conventional immunosuppressants such as cyclophosphamide (CYC) (9–11). The role of newer agents, including interleukin (IL)-5-targeting antibodies, awaits further definition as acute cardiomyopathy was excluded from clinical trials so far (12–14).

Alongside immunosuppression, heart failure (HF) pharmacotherapy is a cornerstone in managing cardiac dysfunction (15). In acute myocarditis in general, and in EGPA and HES in particular, cardiomyopathy with left-ventricular (LV) dysfunction constitutes a negative prognostic factor associated with excess morbidity and mortality (10, 16–18). Although the European Society of Cardiology (ESC) HF guidelines temporarily recommend pharmacotherapy for LV dysfunction in myocarditis, they do not differentiate between distinct etiologies (7, 15). Taken together, as substantial diagnostic and therapeutic uncertainties remain, preserving cardiac function appears crucial, and biomarker-driven therapies offer promising avenues to improve outcomes in EM.

In this study, we aimed to identify prognostic biomarkers that inform future therapeutic decision-making across various underlying IRD by addressing EM as a distinct entity in a real-world cohort. Long-term outcomes of EM were characterized with a focus on cardiac function, integrating clinical patterns, laboratory, and imaging data.

## Materials and methods

### Study design

This single-center retrospective cohort study was conducted at a quaternary referral center in Munich, Germany (Ludwig-Maximilians University Hospital). Electronic medical records were queried for ICD-10-GM (International Classification of Diseases-German Modification) codes M30.1, J82, D47.5, and I42.3, covering EGPA, idiopathic HES (i-HES), and drug-associated/hypersensitivity myocarditis (dEM). Patients treated at our clinic between January 2010 and May 2025 were included if they had an EAD with myocardial involvement and/or biopsy-proven EM. Five patients were diagnosed with EM before 2010 at external institutions; their diagnostic work-up and initial treatment partly predated the study period. Myocardial involvement was defined by fulfilling the 2013 ESC diagnostic criteria for clinically suspected myocarditis and a likely causal relationship with the EAD (Supplementary Table S1) (19). This definition was applied uniformly across all etiologies; however, classification of the underlying EAD was specific to the suspected entity and based on specialist assessment and disease-specific criteria. Drug-associated EM required a plausible clinical and temporal relationship to the suspected exposure and no more likely alternative explanation; idiopathic EM was assigned when neither a systemic eosinophilic disorder nor an alternative trigger could be established. Exclusion criteria were age <18 years or cardiomyopathy due to non-IRD causes (e.g., ischemia, infection, and malignancy).

Clinical, laboratory, and imaging data (transthoracic echocardiography (TTE), CMR, and EMB) were collected. Baseline parameters were acquired before induction therapy. For LV assessment, CMR was preferred due to lower interobserver variability (20); if unavailable, TTE data were used. Qualitative reports of LV ejection fraction (LVEF) were excluded. “Positive” EMB was defined as eosinophilic or active non-eosinophilic (e.g., when acquired after start of immunosuppression (21, 22)) inflammation with/without vasculitis; “positive” CMR was defined as concomitant late gadolinium enhancement (LGE) and myocardial edema. Uncertain or ambiguous findings (e.g., EMB showing fibrosis and isolated LGE) were classified as “inconclusive.” If EMB/CMR was not conducted or unavailable, patients were analyzed separately.

We also applied published composite scores: First, the revised Five-Factor Score (r-FFS; range 0–5) was calculated as an established prognostic score for EGPA (16). Second, a score proposed by Sartorelli et al. in 2022 (range 0–6) was retrospectively calculated at baseline using its predefined original components and point allocation (6). The score was originally developed to identify cardiac involvement in EGPA and comprises clinical, electrocardiographic, biochemical, echocardiographic, and CMR-based features with ≥3 points yielding high sensitivity (85.7%) and specificity (96.4%) in the original study. Its components and scoring algorithm are detailed in Supplementary Table S2. Individual score components were reconstructed from routine-care records. Where source information was not available, the score was considered missing rather than imputed.

No uniform treatment protocol for EM was in place during the study period. Disease-specific recommendations, particularly for severe EGPA, served as a framework for clinical decision-making (8, 23–25). However, these recommendations did not provide an EM-specific treatment algorithm, and immunosuppressive therapy ultimately remained individualized in multidisciplinary routine care, taking into account disease activity, extracardiac involvement, underlying EAD, comorbidities, previous therapy, and year of diagnosis. Recommendations for HES, dEM, and idiopathic EM were even less specific (17). Accordingly, treatment analyses were descriptive and not intended to estimate comparative treatment effects.

This study was approved by the Ethics Committee of the Faculty of Medicine of LMU Munich (24–0417_1) and conducted in accordance with the Declaration of Helsinki and the Professional Code of Conduct for Physicians in Bavaria. Given the retrospective design, patient consent was waived.

### Definition of guideline-concordant HF therapy

Because ESC guidelines for treatment of HF with reduced ejection fraction (HFrEF, LVEF≤40%) evolved substantially over time, “guideline-concordant” HF pharmacotherapy was defined based on class I recommendations applicable during the respective treatment period since 2008 (Supplementary Table S3). The term “HFrEF” was applied to all patients with LVEF ≤ 40% as this threshold remained consistent across guidelines. Drugs included angiotensin-converting enzyme inhibitors, angiotensin receptor blockers, beta-blockers, mineralocorticoid receptor antagonists, sacubitril/valsartan, and sodium-glucose cotransporter 2 inhibitors (15, 26–29). Continued use of previously guideline-concordant therapy was considered appropriate if patients later transitioned to HF categories without class I recommendations.

### Statistics

Descriptive statistics were used to summarize clinical, laboratory, and imaging variables. Normally distributed data were presented as mean ± standard deviation, non-normally distributed or ordinal variables as median and interquartile range (IQR), and categorical data as percentages. Group comparisons used parametric (paired/unpaired *t*-test, one-way analysis of variance (ANOVA), and mixed-effects) or non-parametric tests (Wilcoxon, Mann–Whitney U, Kruskal–Wallis), as appropriate; categorical variables were compared with Fisher’s exact test and correlations with Pearson’s r or Spearman’s rho. Significant overall differences were followed by pairwise *post-hoc* analysis; a Bonferroni correction was applied to account for multiple testing, where appropriate. Linear and logistic regression were applied to assess associations of variables with outcomes, with log_10_-transformation where appropriate. Slopes (β-coefficients) and odds ratios (OR) with 95% confidence intervals (CI), and coefficients of determination (*R*^2^) were reported. Analyses compared initial diagnosis (1Dx), and first and final follow-up (1FU/fFU). 1FU was defined as first routine-care visit with available cardiac evaluation after induction phase. fFU was the last available assessment. Because induction regimens and duration varied substantially, and cardiac assessments were not performed at every clinical visit, no single comparable 1FU timepoint could be identified. A standardized retrospective interim follow-up was not imposed because it would have excluded a substantial proportion of patients without yielding comparable post-induction assessments. If only one follow-up was available, it was analyzed as fFU. Data were obtained within ±3 months of the reference timepoint; when multiple values were available, the most abnormal was selected. If a variable was not documented at 1Dx or fFU, the follow-up with the first or last available variable (First/Last) was used, respectively. Two-sided *p* < 0.05 were considered statistically significant. Analyses were performed with GraphPad Prism 11 (v11.0.0, GraphPad Software, Boston, MA, United States) and supported by the AI-based language model ChatGPT-4o/5.4 Plus (OpenAI, San Francisco, CA, United States).

## Results

### Baseline characteristics and cardiac assessment

Of 419 patients identified through ICD-10-GM coding, 51 with EAD and cardiomyopathy or idiopathic EM fulfilled the ESC criteria for clinically suspected myocarditis and were included in the analysis. Two patients were included based on cardiac relapse while under our care, despite missing baseline data. Table 1 summarizes baseline characteristics. Mean age was 50 ± 14 years, and 51% (26/51) were female. Diagnoses included EGPA (*n* = 29), i-HES (*n* = 16), dEM (*n* = 7), and idiopathic EM (*n* = 3). Among dEM patients, five had eosinophilia associated with the anti-IL-4/13-antibody dupilumab, three of whom had pre-existing EGPA, and one had i-HES. One dEM case each was attributed to cefaclor (a cephalosporin) and ibuprofen. Given the small subgroup sizes of idiopathic EM and dEM, and the partial diagnostic overlap within the latter, formal comparisons were restricted to EGPA and i-HES.

**Table 1:** Baseline characteristics (highest values) of the whole study population and stratified to underlying inflammatory-rheumatic diseases (IRD).

Characteristics	Total (*n* = 51)		EGPA (*n* = 29)		i-HES (*n* = 16)		Idiopathic EM (*n* = 3)		dEM (*n* = 7)		*p* ^a^
	Value	*n* with data	Value	*n* with data	Value	*n* with data	Value	*n* with data	Value	*n* with data	
Age, years	50 ± 14	51	48 ± 15	29	51 ± 15	16	45 ± 12	3	54 ± 11	7	0.80
Female, *n* (%)	26 (51)	51	15 (52)	29	8 (50)	16	2 (67)	3	3 (43)	7	>0.99
EM as first sign of IRD, *n* (%)	11 (23)	47	2 (7)	28	5 (36)	14	n.d.	n.d.	3 (43)	7	**0.03**
EM leading to 1Dx of IRD, *n* (%)	38 (81)	47	23 (82)	28	13 (81)	16	n.d.	n.d.	5 (71)	7	>0.99
Symptoms, *n* (%)											
Chest pain	28 (61)	46	15 (58)	26	8 (57)	14	3 (100)	3	6 (86)	7	>0.99
Dyspnea	34 (76)	45	20 (80)	25	10 (71)	14	2 (67)	3	4 (57)	7	0.70
Obstructive airways	21 (44)	48	17 (63)	27	4 (27)	15	0 (0)	3	1 (14)	7	0.052
Peripheral edema	9 (20)	46	4 (15)	26	4 (29)	14	0 (0)	3	2 (29)	7	0.42
B symptoms^b^	20 (42)	48	10 (37)	27	7 (47)	15	1 (33)	3	3 (43)	7	0.74
Cardiogenic shock	8 (16)	50	4 (14)	28	2 (13)	16	2 (67)	3	0 (0)	7	>0.99
Organ involvement, *n* (%) (at any timepoint)											
Asthma	35 (69)	51	28 (97)	29	7 (44)	16	0 (0)	3	4 (57)	7	**0.0001**
Pulmonary infiltrates	36 (72)	50	23 (82)	28	9 (56)	16	2 (67)	3	4 (57)	7	0.09
Sinonasal abnormalities	35 (69)	51	26 (90)	29	7 (44)	16	1 (33)	3	5 (71)	7	**0.002**
Peripheral nervous system	17 (34)	50	13 (46)	28	3 (19)	16	0 (0)	3	5 (71)	7	0.10
Central nervous system	9 (18)	50	6 (22)	28	3 (19)	16	0 (0)	3	4 (57)	7	>0.99
Laboratory											
Eosinophils, G/L	4.2 [1.5–9.4]	48	4.2 [1.6–8.7]	26	6.7 [3.0–13.3]	16	0.3 [0.02–0.4]	3	3.1 [0.9–4.3]	7	0.14
Eosinophils, %	33 [15–48]	49	35 [16–48]	27	42 [29–53]	16	3 [0–4]	3	29 [13–39]	7	0.33
Troponin T, ng/mL	0.55 [0.08–1.47]	31	0.51 [0.08–1.47]	15	0.54 [0.05–1.67]	11	1.35 [0.77–2.30]	3	0.25 [0.07–0.92]	6	0.89
Troponin I, ng/mL	1.00 [0.45–6.94]	18	4.27 [0.6–21.9]	10	0.89 [0.12–4.18]	7	n.d.	0	0.25	1	0.23
Creatine kinase, U/L	245 [103–517]	46	277 [141–677]	27	182 [97–387]	13	367 [98–1,092]	3	274 [72–566]	7	0.23
(NT-)proBNP, pg/mL	3,403 [2234–11,391]	30	3,931 [2013–11,931]	14	3,345 [2190–8,101]	13	17,665 [2253–22,714]	3	2,779 [1627–8,969]	5	0.91
BNP, pg/mL	761 [153–5,261]	5	834 [107–7,181]	4	761	1	n.d.	0	n.d.	0	n.d.
C-reactive protein, mg/dL	4.5 [2.8–10.3]	49	4.2 [3.1–9.0]	28	4.3 [1.7–10.1]	15	14.3 [14.1–25.2]	3	3.7 [1.0–9.8]	7	0.68
eGFR^c^, mL/min	74 ± 22	47	78 ± 23	27	71 ± 17	14	54 ± 34	3	85 ± 27	7	0.30
ANCA^d^ (any timepoint), *n* (%)	9 (18)	51	7 (24)	29	2 (13)	16	0 (0)	3	2 (29)	7	0.46
Echocardiogram (transthoracic)											
LVEF, %	48 ± 17	36	48 ± 16	22	52 ± 17	9	29 ± 20	3	54 ± 11	5	0.52
LVEDD, mm	50 ± 9	30	51 ± 9	19	50 ± 8	9	41	1	52 ± 7	3	0.90
Pericardial effusion, *n* (%)	24 (53)	45	13 (54)	24	6 (40)	15	2 (67)	3	2 (67)	6	0.51
Cardiac magnetic resonance											
LVEF, %	48 ± 17	29	46 ± 17	16	48 ± 18	10	61	1	57 ± 15	5	0.78
LVESVI, mL/m^2^ BSA	36 [28–62]	25	49 [28–64]	14	35 [27–61]	8	30	1	27 [25–49]	5	0.65
LVEDVI, mL/m^2^ BSA	80 [67–96]	25	83 [67–97]	14	74 [57–97]	8	77	1	75 [70–89]	5	0.47
LGE (any kind), *n* (%)	26 (79)	33	18 (95)	19	6 (55)	11	1 (100)	1	3 (60)	5	**0.02**
Myocardial edema, *n* (%)	17 (61)	28	10 (67)	15	5 (50)	10	1 (100)	1	3 (60)	5	0.44
Pericardial effusion, *n* (%)	18 (62)	29	10 (56)	18	5 (63)	8	1 (100)	1	4 (80)	5	>0.99
Endomyocardial biopsy, *n* (%)											
Active EM	13 (52)	25	4 (36)	11	5 (50)	10	3 (100)	3	1 (50)	2	0.67
Active without eosinophils	4 (16)	25	1 (9)	11	3 (30)	10	0 (0)	3	0 (0)	2	0.31
Post-inflammatory/fibrotic	8 (32)	25	6 (55)	11	2 (20)	10	0 (0)	3	1 (50)	2	0.10
Coronary diagnostics, *n* (%)											
Catheterization performed	38 (75) ^e^^,^^f^	51	20 (69)	29	13 (81) ^e^	16	3 (100)	3	6 (86) ^f^	7	0.49
Coronary artery disease (CAD)	4 (11)	37	1 (5)	20	2 (17)	12	1 (33)	3	0 (0)	6	0.54
Intervention/stenting	1 (2)	51	0 (0)	29	0 (0)	16	1 (33)	3	0 (0)	7	>0.99
Composite scores											
Sartorelli 2022 (0–6 points)	5 [3–5.5]	49	5 [3–6]	27	5 [2.3–5]	16	5 [5–6]	3	4 [2–6]	7	0.33
r-FFS 2011 (0–5 points)	1 [1–2]	49	1 [1–2]	27	2 [1–3]	16	3 [1–3]	3	1 [1–1]	7	0.08

ANCA, anti-neutrophil cytoplasmatic antibodies; BSA, body surface area; dEM, drug-associated/hypersensitivity myocarditis; eGFR, estimated glomerular filtration rate; EGPA, eosinophilic granulomatosis with polyangiitis; EM, eosinophilic myocarditis; i-HES, idiopathic hypereosinophilic syndrome; LGE, late gadolinium enhancement; LVEDD, left-ventricular end-diastolic diameter; LVEF, LV ejection fraction; LVESVI/LVEDVI, LV end-systolic/−diastolic volume indices; n.d., not determined; (NT-)proBNP, (N-terminal) pro-brain-type natriuretic peptide; r-FFS, revised Five-Factor Score; 1Dx, initial diagnosis.

a Comparisons were limited to EGPA and i-HES due to low sample size in other subgroups or diagnosis overlap (dEM).

b Includes fever, night sweats, and unexplained weight loss.

c If eGFR was measured as a range (“>60 mL/min”), “80 mL/min” was used for analysis.

d ANCA included: perinuclear (p)-ANCA (EGPA: *n* = 5; dEM: *n* = 2), cytoplasmic (c)-ANCA (EGPA: *n* = 1), atypical (X)-ANCA (EGPA: *n* = 3; i-HES: *n* = 2; dEM: *n* = 1), myeloperoxidase (MPO)-ANCA (EGPA: *n* = 2).

e One catheterization was carried out during later disease course but showed no CAD.

f Instead of catheterization, one patient received coronary computed tomography angiography that showed no CAD.

In 81% (38/47), cardiomyopathy led to diagnosis of the underlying IRD, although it was the first symptom in only 23% (11/47). Compared to i-HES, cardiomyopathy was less frequent a first sign of EGPA [7% versus (vs.) 36%, *p* = 0.03]. Common presenting symptoms were dyspnea (76%, 34/45) and chest pain (61%, 28/46). Pulmonary infiltrates (72%, 36/50), asthma (69%, 35/51), and sinonasal abnormalities (69%, 35/51) were frequent, with the latter two occurring more often in EGPA than i-HES (97% vs. 44%, *p* = 0.0001; 90% vs. 44%, *p* = 0.002).

Laboratory parameters did not differ significantly between subgroups. Peripheral eosinophilia was present in 84% (41/49) (4.2 [IQR 1.5–9.4] G/L; 33 [15–48]% of whole blood count). All 45 patients with available data had elevated high-sensitive troponin T (0.55 [0.08–1.47] ng/mL, normal: <0.014 ng/mL) or troponin I (1.00 [0.45–6.94] ng/mL, normal: <0.02–0.06 ng/mL). In total, 94% (32/34) showed elevated N-terminal pro-brain-type natriuretic peptide (NT-proBNP) (3,403 [2,234–11,391] pg./mL) or BNP. Infrequent detection of myeloperoxidase anti-neutrophil cytoplasmatic antibodies (MPO-ANCA) in EGPA patients (2/29) was consistent with known patterns in cardiac EGPA (6).

**Figure 1:**
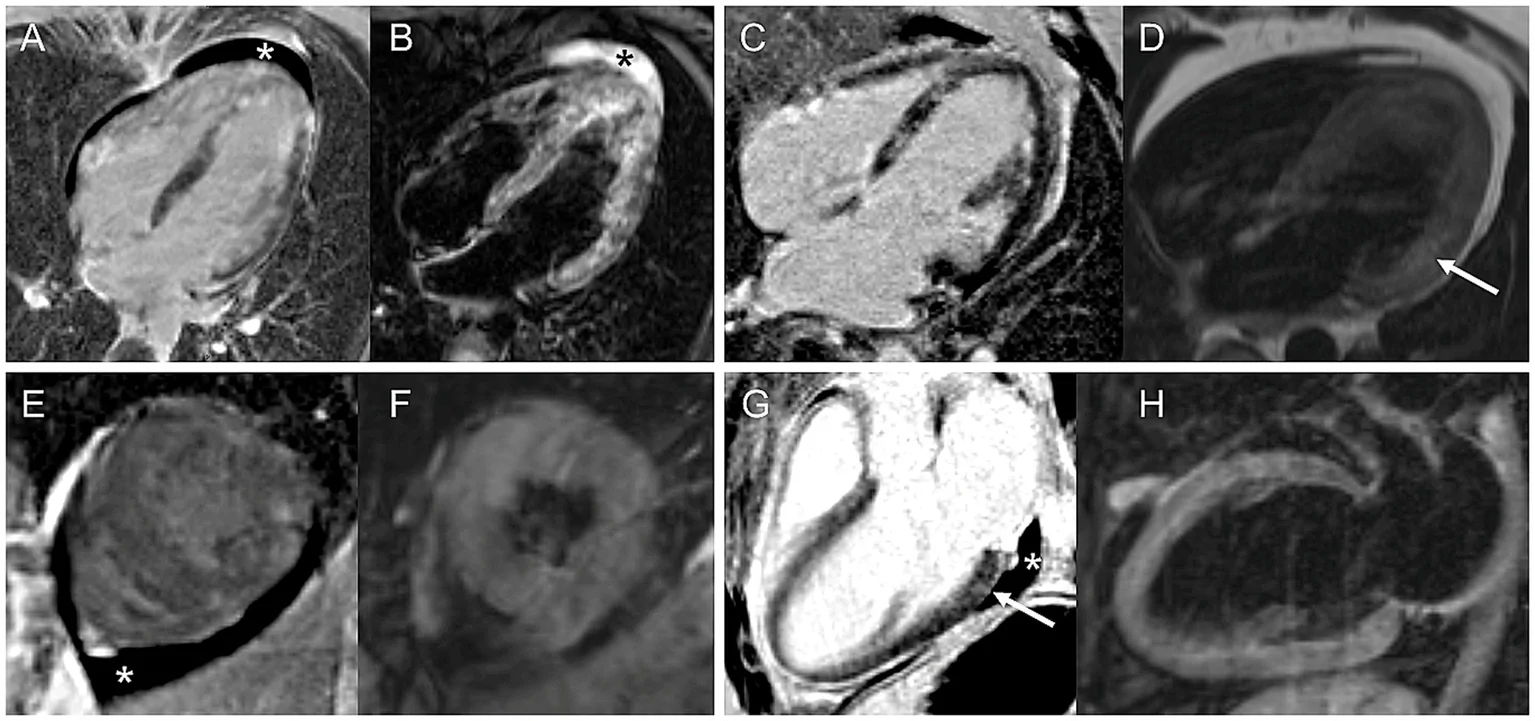
Cardiac magnetic resonance findings in acute EM across eosinophil-associated disorders. Upper left panels: Four-chamber phase-sensitive inversion recovery (PSIR) image **(A)** of a patient with eosinophilic granulomatosis with polyangiitis demonstrates patchy subendocardial, mid-myocardial, and subepicardial late gadolinium enhancement (LGE) involving both ventricles. Corresponding myocardial edema is evident on the T2-weighted image **(B)**, with an associated pericardial effusion (asterisk). Upper right panels: Cardiac magnetic resonance (CMR) in a patient with hypereosinophilic syndrome demonstrates biventricular LGE with subendocardial predominance **(C)**. Myocardial edema is particularly evident in the basal lateral wall of the left ventricle **(D**, arrow**)**. Lower left panels: Short-axis CMR images of a patient with hypersensitivity/cephalosporin-associated EM demonstrate patchy subendocardial, mid-myocardial, and subepicardial LGE in the left ventricle, with predominant subendocardial involvement of the right ventricle **(E)** and semi-circumferential pericardial effusion on PSIR imaging (asterisk). The T2-weighted image **(F)** reveals diffuse myocardial edema with anterior wall predominance. Lower right panels: CMR imaging of a patient with idiopathic EM. The three-chamber PSIR view **(G)** demonstrates subtle subepicardial LGE in the basal inferolateral left ventricular wall (arrow) with adjacent small pericardial effusion (asterisk). Despite respiratory motion artifacts, the two-chamber T2-weighted image **(H)** is suggestive of patchy myocardial edema.

Mean LVEF was 48 ± 17% (*n* = 42) without subgroup differences. TTE and CMR results were comparable (*r* = 0.54; *p =* 0.007); 16% (8/50) had cardiogenic shock; 45% (22/49) and 12% (6/49) were treated in an intensive and intermediate care unit, respectively; 13% (6/48) received inotropes, and 6% (3/48) required temporary mechanical circulatory support. Seven patients were excluded from LVEF analysis due to qualitative LVEF reporting (“normal”: *n* = 5, “mildly reduced”: *n* = 1, “mild-to-moderately reduced”: *n* = 1). Among imaging parameters, only LGE (of any kind) was more frequent in EGPA than i-HES (95% vs. 55%, *p* = 0.02). LV diameter and volume were within the normal range in most patients. Twenty-five patients (49%) underwent EMB: Active eosinophilic, active non-eosinophilic, and post-inflammatory/fibrotic lesions were seen in 52, 16, and 32%, respectively. Overall, 47% (24/51) had any “positive” and 29% (15/51) any “inconclusive” EMB/CMR findings; in the remaining 24% (12/51), no EMB/CMR was performed or results were unavailable. Representative “positive” CMR images illustrating features of acute EM across EGPA, i-HES, dEM, and idiopathic EM are shown in Figure 1.

Median r-FFS and Sartorelli score were 1 [1–2] and 5 [3–5.5], respectively; 82% (40/49) had a Sartorelli score of ≥3. Coronary angiography was performed in 75% (38/51) with only one patient requiring intervention. This patient remained in the analysis since EMB unambiguously showed fulminant EM.

### Disease course and outcomes

Data of first follow-up (1FU) were available after a median of 20 [8–36] weeks; final follow-up (fFU) covered 2,877 patient-months (median 34 [16–79] months). As no uniform EM treatment protocol was in place, induction regimens reflected individualized multidisciplinary decisions. Individual induction strategies are detailed in Supplementary Table S4. In the overall cohort, induction therapy included GC in 96% (48/50) with a median starting dose of 200 [68–1,000] mg prednisone equivalent/day (2.3 [1–10] mg/kg/day); 59% (27/46) received GC pulses at a median dose of 500 [250–1,000] mg/day for a median of 3 days. GC tapering to <7.5 mg/day took 190 [110–526] days, and 22 [5–56] months until withdrawal. CYC was used in 16 patients (mean cumulative induction dose 6,320 ± 1,754 mg). Other immunosuppressants initiated at 1Dx included mepolizumab (*n* = 5), benralizumab (*n* = 3), rituximab (*n* = 3), azathioprine (*n* = 2), and non-steroidal anti-inflammatory drugs/colchicine (*n* = 10).

**Figure 2:**
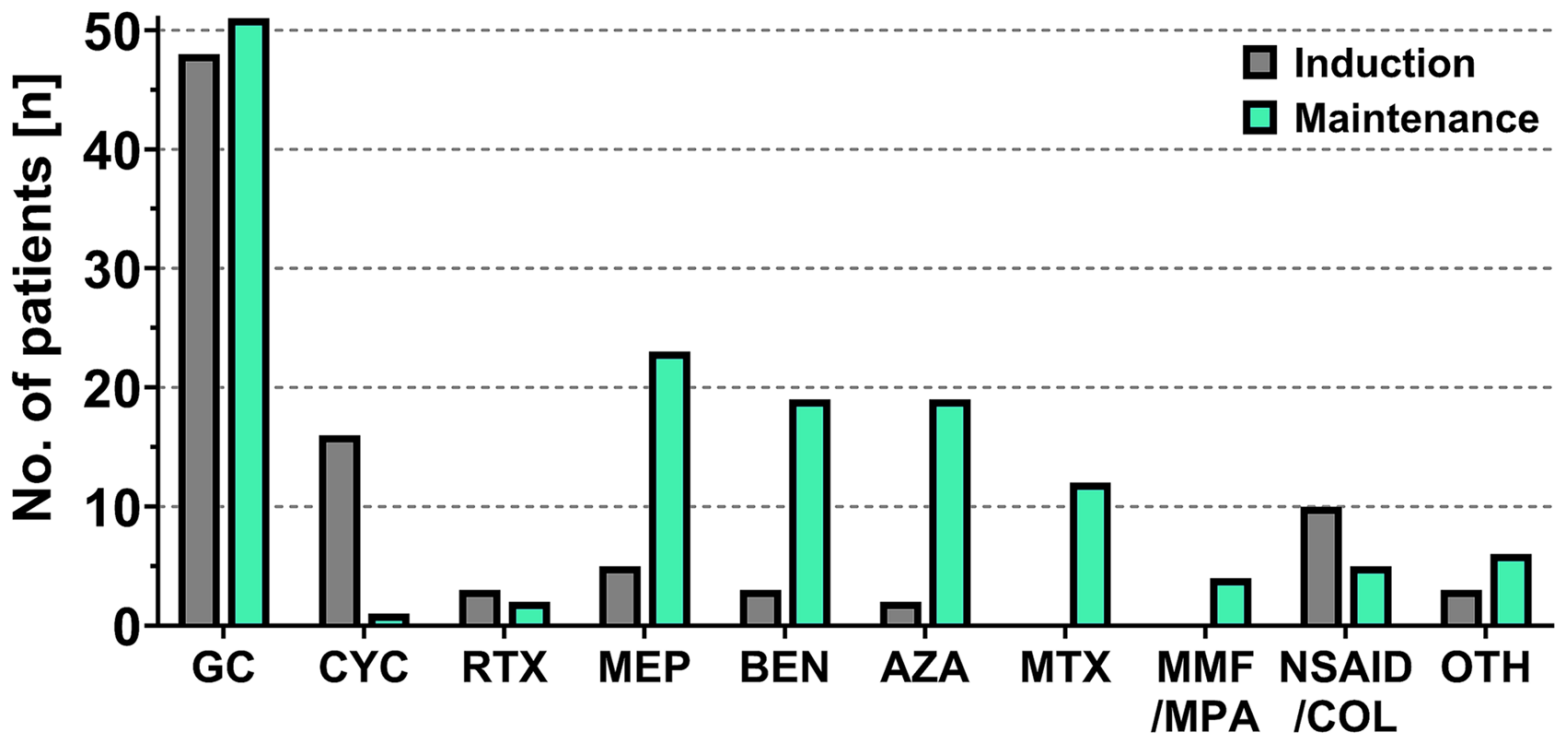
Immunomodulatory agents applied in EM patients. AZA, azathioprine; BEN, benralizumab; CYC, cyclophosphamide; GC, glucocorticoids; MEP, mepolizumab; MMF/MPA, mycophenolate(−mofetil); MTX, methotrexate; NSAID/COL, non-steroidal anti-inflammatory drug/colchicine; OTH, other agents (immunoglobulins, hydroxyurea, cyclosporin a, interferon alpha, reslizumab, and tezepelumab); RTX, rituximab. Some patients received more than one agent.

Maintenance therapy included GC in all cases. Most patients received more than one additional agent, most commonly mepolizumab (*n* = 23), benralizumab (*n* = 19), azathioprine (*n* = 19), and methotrexate (*n* = 12; Figure 2).

At fFU, 64% (32/50) were free of cardiac symptoms. 90% (45/50) continued immunomodulatory drugs; 54% (27/50) were taking GC (5 [2.5–7.5] mg/day after a median of 34 months). Five patients died: three from infections likely related to the underlying IRD or therapy and two from EM-related HF. Deaths occurred both early and late after diagnosis (two within the first year, one each at 2, 3, and 8 years).

**Figure 3:**
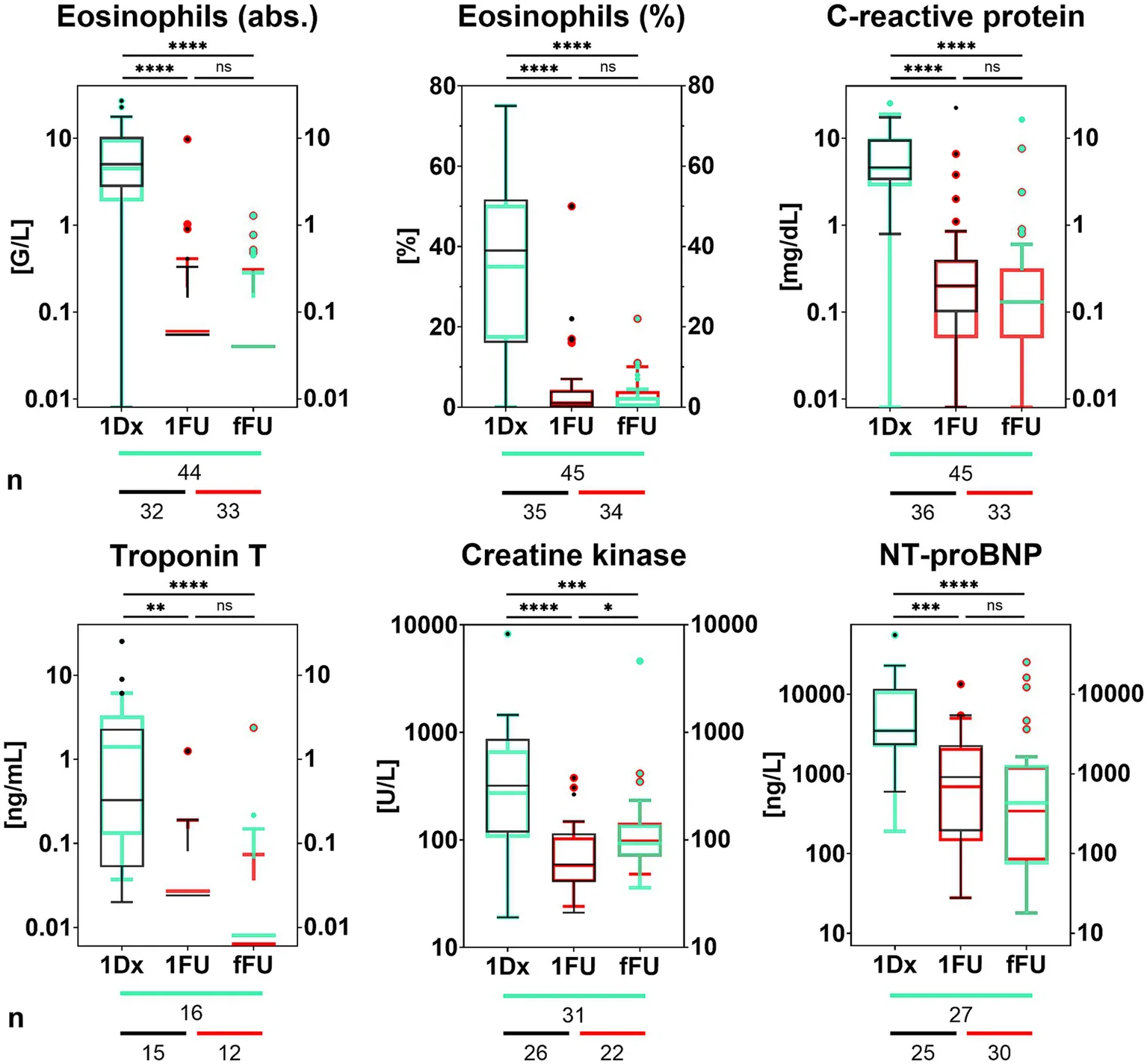
Course of laboratory parameters in EM patients illustrated by Tukey’s boxplots. Due to missing values at individual timepoints, comparisons were performed pairwise using the Wilcoxon signed-rank test; a Bonferroni correction was applied to account for multiple testing. Different colors account for paired data (green: 1Dx-fFU, black: 1Dx-1FU, red: 1FU-fFU); *n* refers to number of pairs. 1Dx, initial diagnosis; 1FU, first follow-up; fFU, final follow-up; NT-proBNP, N-terminal pro-brain-type natriuretic peptide; box, interquartile range (IQR); horizontal line: median; whiskers: 1.5 × IQR; points: outliers. ns, not significant; *p < 0.05; **p < 0.01; ***p < 0.001; ****p < 0.0001.

Laboratory parameters improved significantly from 1Dx to 1FU and from 1Dx to fFU, but not between 1FU and fFU (Figure 3). At fFU, median absolute/relative eosinophil counts (AEC/REC), troponin, creatine kinase (CK), and C-reactive protein (CRP) had normalized, whereas NT-proBNP remained elevated in 46% (19/41; median 421 [80–1,248] pg/mL; Supplementary Figure S1).

**Figure 4:**
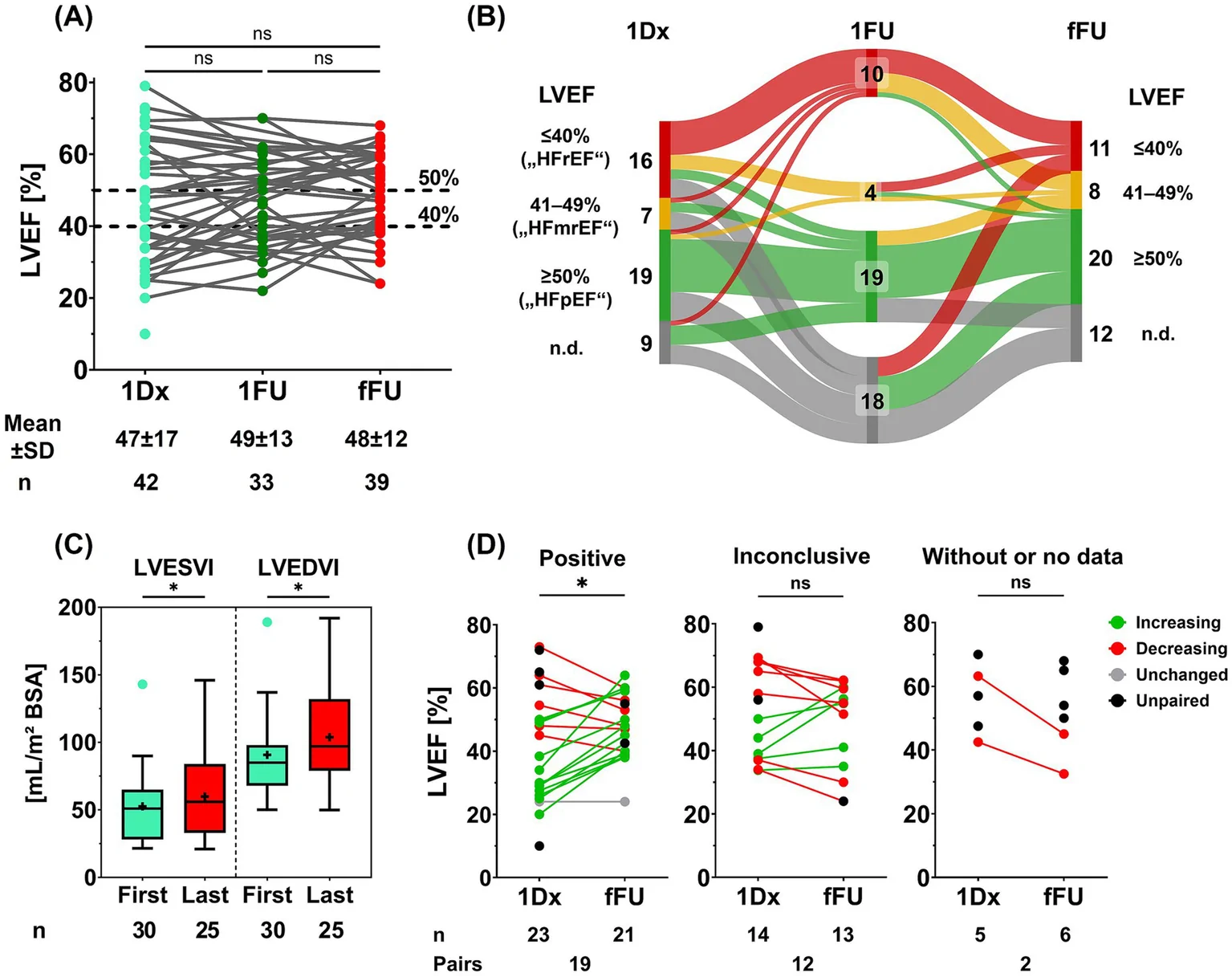
Course of left-ventricular ejection fraction (LVEF) and volume indices in EM patients. **(A)** Individual LVEF course of every patient with available data. *n*(pairs) = 28–33. **(B)** Transition of LVEF-groups over time defined by thresholds of the European Society of Cardiology. HFr/mr/pEF, heart failure with reduced/mildly reduced/preserved ejection fraction. Sankey plot was generated using SankeyMATIC (v.2023-11-11). **(C)** Course of LV end-systolic and end-diastolic volume indices (LVESVI/LVEDVI) stratified to body surface area (BSA) via CMR. First/Last, first/last available values. Box = interquartile range (IQR); horizontal line = median; whiskers = 1.5 × IQR; points = outliers; cross = mean; *n*(pairs) = 19. **(D)** LVEF course stratified to baseline findings in endomyocardial biopsy (EMB) or cardiac magnetic resonance (CMR). Positive = eosinophilic or active non-eosinophilic inflammation (with/without vasculitis) in EMB and/or presence of both late gadolinium enhancement and myocardial edema in CMR. Inconclusive = incomplete or ambiguous findings. Without/no data = EMB/CMR not conducted or data unavailable. 1Dx, initial diagnosis; 1FU, first follow-up, fFU, final follow-up, n.d., not determined. Mean ± standard deviation (SD) and *n* refers to all individuals at the respective timepoint (including unpaired values) statistical analysis refers to paired values only. ns, not significant. * *p* < 0.05.

LVEF showed no significant improvement over time (1FU: *Δ* + 0.6 ± 11%; fFU: Δ + 3 ± 12%, *n* = 28–33 pairs; *p* = 0.57), and mean LVEF was reduced after a median of 34 months (48 ± 12%, *n* = 39). (Figure 4A). TTE and CMR results remained comparable (*r* = 0.85; *p* = 0.0004). Of patients with baseline LVEF<50% and available data, only 29% (6/21) improved to a “normal” LVEF range (i.e., 50–70%) (15).

Median LV volumes increased over time: LV end-systolic volume index (LVESVI) increased from 36 [28–62] to 56 [31–74] mL/m^2^ body surface area (BSA) at 1FU and to 51 [29–101] mL/m^2^ at fFU. LV end-diastolic volume index (LVEDVI) rose from 80 [67–96] to 101 [71–121] and 93 [68–140] mL/m^2^ BSA, respectively. Paired comparison (*n* = 19) of first-available and last-available values confirmed a significant increase of LVESVI (50 [28–65] to 56 [33–84] mL/m^2^; *p* = 0.037) and LVEDVI (85 [68–98] to 97 [79–132] mL/m^2^; *p* = 0.016) (Figure 4C). To explore the clinical relevance of LV dilatation, associations with cardiac outcomes were analyzed. Absolute increases of ≥5 mL/m^2^ in LVESVI or LVEDVI between first and last available CMR were not associated with NT-proBNP levels, cardiac symptoms, rhythm-related events, or mortality at fFU. Rhythm-related events comprised documented arrhythmias or conduction abnormalities—including ventricular fibrillation or tachycardia, atrial fibrillation or flutter, and bundle branch block—as well as clinically reported palpitations. However, higher LVESVI and LVEDVI at last examination were each associated with higher log_10_-transformed NT-proBNP levels at fFU in age- and sex-adjusted multivariable linear regression analyses (LVESVI: *r* = 0.7; *β* = 0.15 [0.09–0.22], *R*^2^ = 0.61; LVEDVI: *r* = 0.62, *β* = 0.12 [0.06–0.19], *R*^2^ = 0.5; all *p* ≤ 0.0014) (Supplementary Figure S2A). In multivariable logistic regression analyses, LV volumes were also associated with any cardiac symptoms (LVESVI: OR 1.5 [1.1–2.3] per +10 mL/m^2^; LVEDVI: OR 1.4 [1.1–2.1] per +10 mL/m^2^) and rhythm-related events at fFU (LVESVI: OR 1.5 [1.1–2.3] per +10 mL/m^2^; LVEDVI: OR 1.4 [1.1–2.0] per +10 mL/m^2^) (Supplementary Figure S2B). Given the low number of deaths, mortality was not analyzed.

Finally, analyses were stratified by baseline EMB/CMR findings, acknowledging that EM is frequently diagnosed without confirmatory EMB/CMR in routine care (30, 31). Patients with “positive” EMB/CMR exhibited greater disease burden compared to “inconclusive” EMB/CMR reflected by higher Sartorelli score (5 [4–6] vs. 3 [2–5], *p* = 0.04), r-FFS (2 [1–3] vs. 1 [0–1], *p* = 0.006), and numerically lower LVEF (43 ± 18% vs. 53 ± 15%; *p* = 0.12). Despite this, only patients with “positive” EMB/CMR showed significant mean LVEF improvement at fFU (41 ± 15 to 48 ± 10%; *p* = 0.017 for pairs) (Figure 4D). Compared to inconclusive findings, positive baseline EMB/CMR was associated with higher odds of ≥5%-LVEF improvement at fFU (OR 7.6 [1.4–61.4]). NT-proBNP improved in all EMB/CMR subgroups, whereas the “inconclusive” group exhibited the largest numerical increase in LVESVI/LVEDVI. Treatment regimens did not differ (Table 2).

**Table 2:** Parameters of cardiac function stratified to baseline endomyocardial biopsy (EMB) and cardiac magnetic resonance imaging (CMR) findings.

Characteristics	Time point	Positive		Inconclusive		Without EMB/CMR or no data (*n* = 12)		*p*
		EMB/CMR (*n* = 24)		EMB/CMR (*n* = 15)				
		Value	*n* with data	Value	*n* with data	Value	*n* with data	
(NT-)proBNP, pg/mL	1Dx	3,577 [2551–15,089]	17	3,232 [1165–7,692]	9	2,379 [1531–5,821]	4	0.22
	fFU	481 [81–2,142]	22	260 [90–1,076]	13	84 [72–1,521]	5	0.74
LVEF, %	1Dx	43 ± 18	23	53 ± 15	14	56 ± 11	5	0.12
	fFU	48 ± 10	21	47 ± 15	13	52 ± 13	6	0.68
LVESVI, mL/m^2^ BSA	First	35 [29–63]	16	43 [24–71]	12	29 [27–31]	2	0.53
	Last	56 [31–78]	14	88 [43–114]	6	37 [25–47]	5	0.10
LVEDVI, mL/m^2^ BSA	First	76 [67–92]	16	89 [64–122]	12	62 [50–74]	2	0.24
	Last	92 [67–124]	14	128 [100–168]	6	83 [65–95]	5	0.06
Sartorelli score 2022, (0–6 points)	1Dx	5 [4–6] ^1^	24	3 [2–5] ^2^	14	3 [2–5] ^3^	11	0.02 (global) ^1 vs.2^ 0.04 ^1 vs. 3^ 0.12 ^2 vs. 3^ > 0.99
r-FFS 2011, (0–5 points)	1Dx	2 [1–3]	24	1 [0–1]	15	1 [1–2]	10	0.007 (global) ^1 vs.2^ 0.006 ^1 vs. 3^ 0.34 ^2 vs. 3^ 0.78
Therapy								
Glucocorticoids, mg/d	IT	250 [80–1,000]	24	200 [60–1,000]	13	100 [64–400]	8	0.52
Glucocorticoids, mg/kg/d	IT	3 [1–12]	24	2 [1–10]	13	1 [0.8–1.8]	7	0.34
Cyclophosphamide, *n* (%)	IT	8 (33)	24	4 (27)	15	4 (33)	12	0.80
Cyclophosphamide, mg (cum.^a^)	IT	6,396 ± 1,461	8	6,000 ± 0	2	6,328 ± 2,876	4	0.97
Anti-IL-5, *n* (%)	MT	15 (63)	24	11 (73)	14	5 (45)	11	0.41
Guideline-concordant heart failure therapy, *n* (%) [% of recommended target dose]	1Dx	6 (55) [38 ± 22]	11	2 (40) [30 ± 18]	5	n.d.	0	>0.99
	fFU	4 (67) [66 ± 24]	6	3 (75) [46 ± 25]	4	1 (100) [53]	1	>0.99

1Dx, initial diagnosis; anti-IL-5, anti-interleukin-5 monoclonal antibody (i.e., mepolizumab and benralizumab); fFU, final follow-up; First/Last, First/Last available values; IT, induction therapy; LVEF, left-ventricular ejection fraction; LVESVI/LVEDVI, LV end-systolic/−diastolic volume indices; MT, maintenance therapy; n.d., not determined; (NT-)proBNP, (N-terminal) pro-brain-type natriuretic peptide; r-FFS, revised Five-Factor Score.

Mean values of all patients (including unpaired data) are indicated; comparative analysis of LVEF change between 1Dx and fFU was limited to patients with data at both timepoints.

a Refers to cumulative cyclophosphamide dose after completion of induction.

### Heart failure pharmacotherapy

According to the ESC thresholds for HF with reduced, mildly reduced, and preserved ejection fraction, 38, 17, and 45% of patients with available baseline data (*n* = 42) had an LVEF of “≤40%,” “41–49%,” and “≥50%,” respectively (15). Corresponding LVEF distributions were 30, 12, and 58% at 1FU and 28, 21, and 51% at fFU, respectively (Figure 4B).

Among patients with baseline LVEF≤40%, 50% (8/16) received HF therapy according to class I recommendations in effect at that time, increasing to 73% (8/11) at fFU. Target dose achievement (15, 27, 28) improved from 36 ± 20% to 57 ± 23%. Mean LVEF improvement in patients treated at both 1Dx and fFU (6/16) was +11 ± 10%, compared with +0.6 ± 0.8% in those treated only at 1Dx (2/16); three untreated patients improved by +21 ± 8%. Among cases with persistent LVEF≤40%, 75% (6/8) received recommended therapy at 1Dx and fFU individually but only 50% at both timepoints. Target dose achievement improved from 33 ± 20% to 57 ± 27%.

Immunosuppressive regimens did not significantly differ between LVEF subgroups. At fFU, cardiac symptoms were more common in patients with baseline LVEF ≤ 40% or 41–49% than in those with LVEF ≥ 50% (63 and 57% vs. 11%; *p* = 0.002). All deaths occurred in patients with baseline LVEF < 50% (Supplementary Table S5).

### Prognostic biomarkers of cardiac function

Since cardiac function is prognostically relevant in acute myocarditis in general, we specifically examined associations between baseline parameters and longitudinal cardiac outcomes in EM.

**Figure 5:**
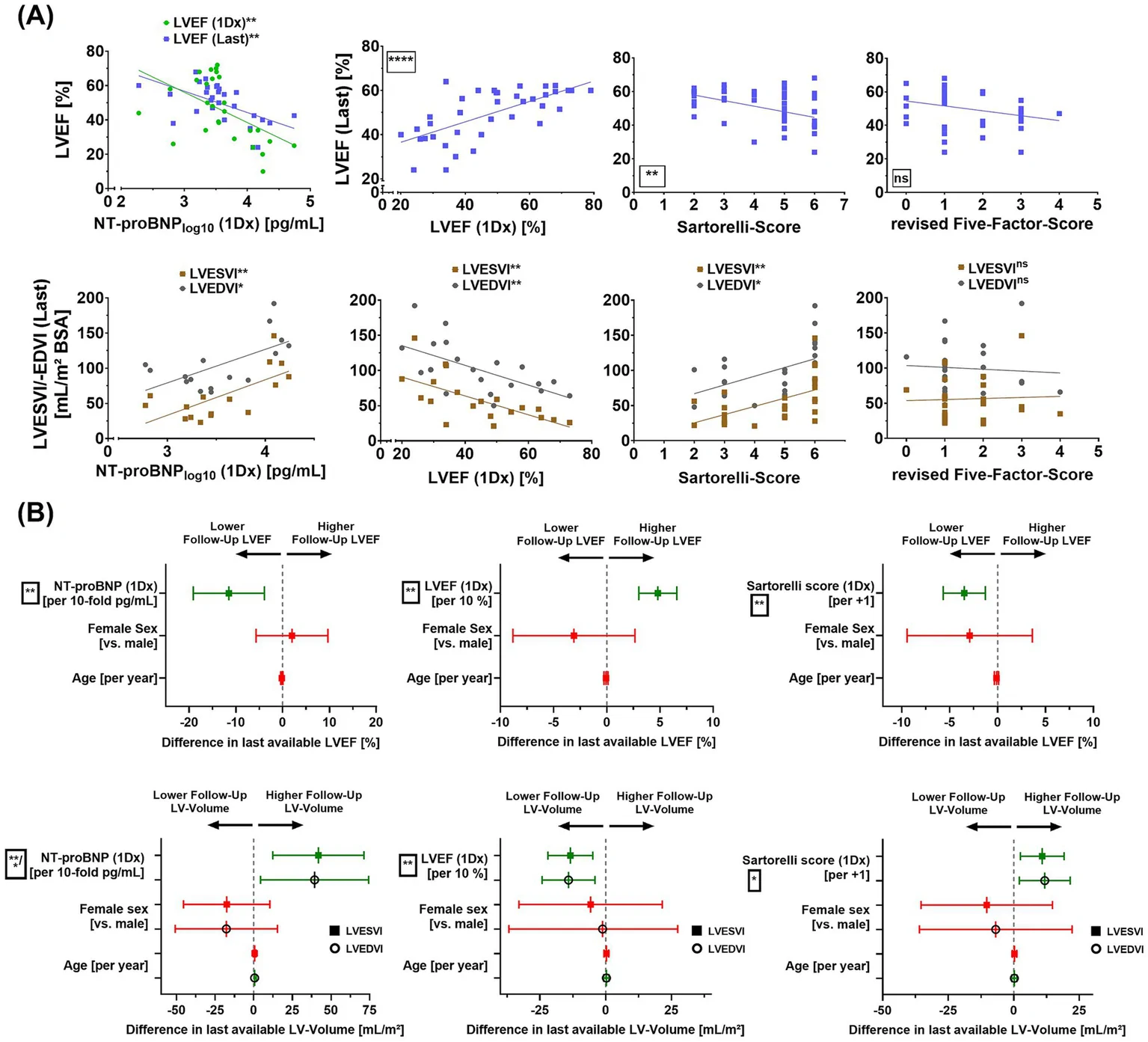
Association of baseline biomarkers with cardiac function in EM patients. **(A)** Univariable linear regression; straight line: Simple linear regression line. Baseline biomarkers are plotted on the *x*-axis. **(B)** Multivariable linear regression depicting slopes (*β*) with 95% confidence intervals. Baseline biomarkers are listed on the *y*-axis, while the *x*-axis indicates the estimated cardiac outcome difference. 1Dx, initial diagnosis; BSA, body surface area; Last, last available values; LVEF, left-ventricular ejection fraction; LVESVI/LVEDVI, LV end-systolic/diastolic volume indices. *N*-terminal pro-brain-type natriuretic peptide (NT-proBNP) includes proBNP and was log_10_-transformed for statistical analysis. ns, not significant; *p < 0.05; **p < 0.01; ****p < 0.0001.

Baseline NT-proBNP levels (log_10_-transformed) were inversely associated with both baseline and last-available LVEF (*β*_1Dx_ = −17.7 [95% CI: −30 to −5.3], *R*^2^_1Dx_ = 0.26, *β*_Last_ = −12.5 [−19.8 to −5.1], *R*^2^_Last_ = 0.32) and positively with last-available LVESVI (*β* = 50.5 [19.2–81.7], *R*^2^ = 0.46) and LVEDVI (*β* = 47.6 [13.1–82.2], *R*^2^ = 0.38, all *p* ≤ 0.01) (Figure 5A). Correlation analysis confirmed these findings (|*r*| = 0.51–0.68; *p* ≤ 0.01). Eosinophil counts (log_10_-transformed) and troponin T levels showed weak associations with baseline LVEF only (e.g., r_AEC_ = 0.37, *β*_AEC_ = 7.1 [1.3–12.8], *R*^2^_AEC_ = 0.14) (*p* = 0.017–0.069) but not with follow-up LVEF or LV volumes. CK and CRP were not predictive (Supplementary Figure S3). Baseline LVEF itself was significantly associated with cardiac outcomes. It correlated positively with last-available LVEF (*r* = 0.67) and inversely with LVESVI (rho = −0.72) and LVEDVI (rho = −0.6). Linear regression analyses confirmed its predictive value: Baseline LVEF predicted follow-up LVEF (*β* = 0.46 [0.29–0.64], *R*^2^ = 0.45), LVESVI (*β* = −1.3 [−2.1 to −0.6], *R*^2^ = 0.42), and LVEDVI (*β* = −1.4 [−2.3 to −0.5], *R*^2^ = 0.38; all *p* ≤ 0.004) (Figure 4A). The Sartorelli score was also associated with last-available LVEF (rho = −0.44; *β* = −3.3 [−5.4 to −1.2], *R*^2^ = 0.19), LVESVI (rho = 0.58; *β* = 11.7 [3.6–19.8], *R*^2^ = 0.29) and LVEDVI (rho = 0.56; *β* = 12.4 [3.2–21.6], *R*^2^ = 0.26; all *p* ≤ 0.01), whereas the r-FFS was not (*p* = 0.083–0.88) (Figure 5A). After adjustment for age and sex in a multivariable regression model, baseline LVEF, NT-proBNP, and Sartorelli score all remained significantly associated with follow-up LVEF (*R*^2^ = 0.23–0.47; *p* < 0.01) and volumetric indices (*R*^2^ = 0.29–0.64; *p* < 0.05) (Figure 5B). In summary, LVEF, log_10_-transformed NT-proBNP, and the Sartorelli score were the only baseline variables consistently associated with all three subsequent cardiac outcomes, namely, last-available LVEF, LVESVI, and LVEDVI, whereas positive baseline EMB/CMR findings were associated with LVEF improvement.

## Discussion

This single-center retrospective study provides real-world evidence on myocardial involvement in eosinophilic disorders. We confirm the prognostic value of biomarkers for long-term cardiac function in inflammatory-rheumatic EM and provide novel insights into diagnosis, treatment, and outcomes. Because EM diagnosis in clinical practice is challenging, and delay of treatment can be fatal, biomarkers that enable risk-adapted care are of particular interest.

Our data were obtained from a representative cohort. EGPA, i-HES, and hypersensitivity were the predominant etiologies; patients were typically middle-aged and presented with dyspnea or chest pain (1, 5, 32). Four patients with drug-associated EM had underlying EGPA or i-HES, complicating distinction from disease flares. Given the temporal relationship and improvement upon drug withdrawal, the cases were classified as such. Peripheral eosinophilia was absent in 16%, supporting earlier findings that up to 25–43% of EM patients have normal eosinophil counts (1, 33). Troponin and NT-proBNP were elevated in nearly all patients, confirming their role in EAD (6). Coronary artery disease was not considered causative in any case, supporting an individualized rather than routine invasive approach guided by pretest probability and less common cardiac manifestations such as coronary vasculitis (7, 34, 35).

At diagnosis, 55% had reduced LVEF<50% and 16% cardiogenic shock, indicating more severe cardiac dysfunction than previously reported (5, 6), whereas long-term mortality was lower (1, 18, 32). Recovery was often incomplete: NT-proBNP remained elevated in nearly 50%, and >70% of patients with LVEF<50% had plateauing or deteriorating LVEF after a median of 34 months. Although LV dimensions at baseline were largely not increased, consistent with an acute presentation, LV dilated over time (33, 36). This suggests adverse remodeling and that LVEF alone may not fully capture persistent structural consequences of EM. Structural remodeling—particularly LV dilatation—has been associated with an increased risk of arrhythmic events and adverse clinical outcomes, including cardiac death, hospitalization, and incident HF, in both inflammatory and non-inflammatory cardiomyopathies, even among patients with preserved LVEF at baseline (37–41). In murine autoimmune myocarditis, progression to dilated cardiomyopathy depended on eosinophils, implicating them in disease pathophysiology (42). Our findings add clinical evidence for progressive LV dilation in EM and support previous data linking increased LVESVI to myocardial EGPA (43). Higher absolute LVESVI and LVEDVI were associated with NT-proBNP levels, residual cardiac symptoms, and rhythm-related events. The latter endpoint—comprising atrial and ventricular arrhythmias, conduction abnormalities and patient’s report of palpitations—was intentionally broad because 12-lead ECG or Holter ECG monitoring were not uniformly available in this retrospective cohort. Inclusion of palpitations aimed to capture arrhythmias on the symptom level. Although causality cannot be inferred and these findings should be considered hypothesis-generating, they indicate that persistent LV dilatation contributes to residual cardiac burden in EM.

No biomarkers are currently validated to predict cardiac function in EAD. NT-proBNP and baseline LVEF are well-established biomarkers in myocarditis and HF in general (4). In this study, we corroborate their relevance specifically in EM. Both predicted long-term LVEF and LV remodeling (38), and all deaths occurred in patients with reduced baseline LVEF, underscoring the prognostic value of cardiac baseline function (33). We additionally found that the recently published Sartorelli score was associated with follow-up LVEF and LV dimensions and outperformed the widely used prognostic r-FFS. As the score was originally developed to detect cardiac involvement in EGPA rather than to predict long-term outcomes in a mixed EAD population, findings related to this score should be considered exploratory. Prospective external validation, particularly outside EGPA, is required before this score can inform routine care. Nevertheless, its performance in our cohort suggests potential prognostic value that warrants further investigation. Its individual components reflect standard cardiac assessment domains. In addition to LV dysfunction, arrhythmias, and CMR findings, the score incorporates serum troponin, which outperformed NT-proBNP for diagnostic accuracy in the original study but was not predictive in our cohort (6). Whether replacing troponin with NT-proBNP, or applying the score at different disease stages, improves its prognostic utility remains to be determined.

A key discovery of our study is that significant LVEF improvement occurred predominantly in patients with baseline EMB/CMR findings indicative of acute inflammatory disease. Because overall treatment differences were insignificant, closer monitoring, earlier intervention, or greater reversibility of acute inflammatory lesions may have contributed. Fibrotic lesions within the “inconclusive” EMB/CMR group, which may already occur in early EM (33), are unlikely to be reversible and limit chances of improvement. Furthermore, lower baseline LVEF in EMB-positive patients may reflect indication bias as EMB is often reserved for severe or hemodynamically unstable cases (7, 32). Nonetheless, our data suggest that despite persistently impaired mean LVEF, targeting EM during the acute phase may improve long-term recovery.

**Figure 6:**
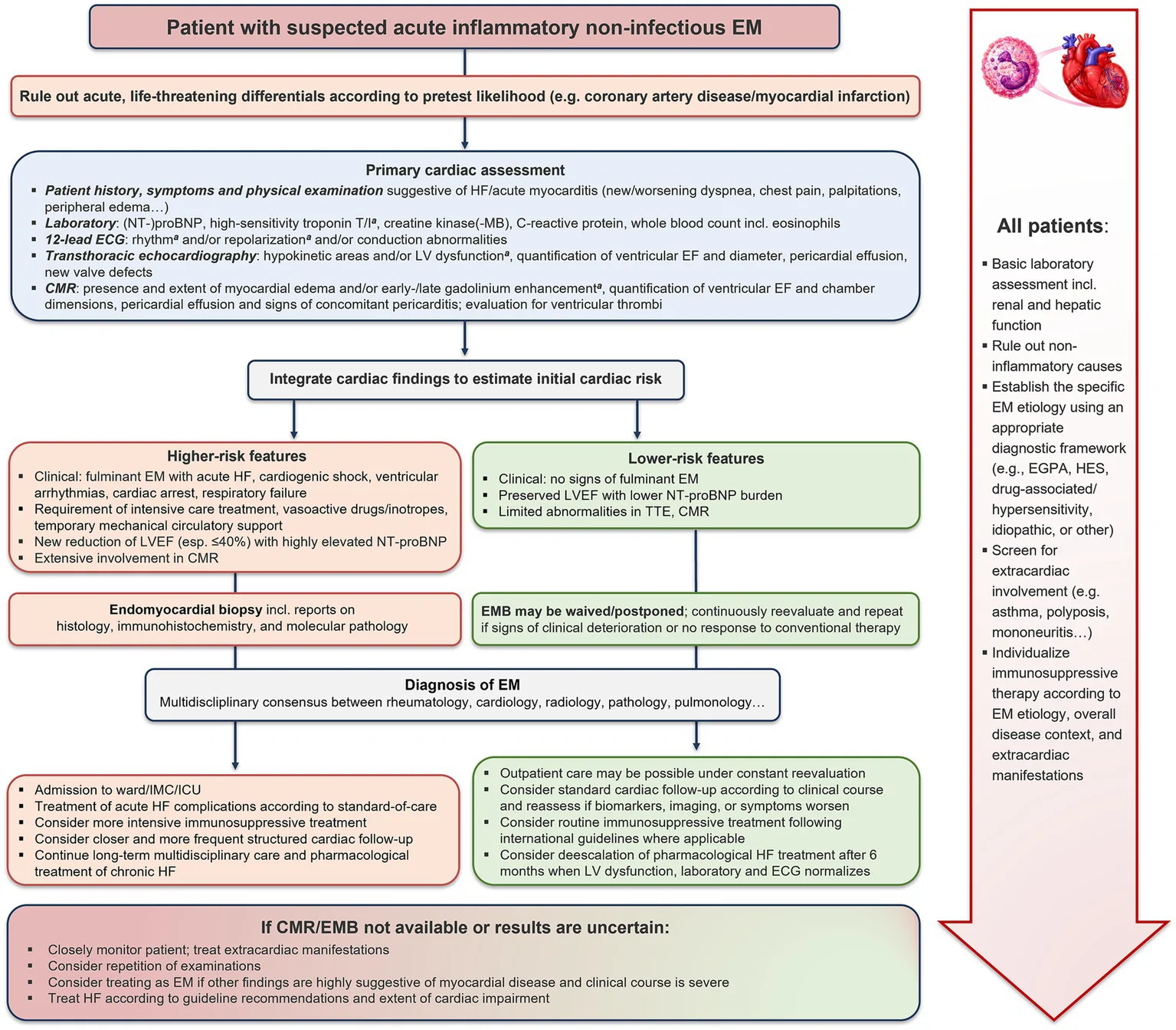
Clinical algorithm for cardiac baseline assessment and risk stratification in eosinophilic myocarditis. This figure provides an author-proposed framework for cardiac assessment and risk stratification in EM and does not constitute a validated diagnostic or treatment algorithm. In the absence of EM-specific guidelines, the framework was informed by the 2025 ESC Guidelines for the management of myocarditis and pericarditis (4) and the 2021 ESC Guidelines for the diagnosis and treatment of acute and chronic heart failure (15). Diagnostic evaluation for the underlying eosinophil-associated disorder should proceed in parallel with cardiac assessment. However, given the potentially life-threatening nature of EM, diagnostic and therapeutic decisions are time-sensitive. Urgent cardiac stabilization and treatment should therefore not be delayed while awaiting completion of all non-cardiac investigations; non-urgent assessments may be postponed until the patient has achieved clinical and hemodynamic stability. CMR, cardiac magnetic resonance; ECG, electrocardiogram; EGPA, eosinophilic granulomatosis with polyangiitis; EM, eosinophilic myocarditis; EMB, endomyocardial biopsy; HF, heart failure; HES, hypereosinophilic syndrome; ICU/IMC, intensive/intermediate care unit; LVEF, left-ventricular ejection fraction; (NT-)proBNP, (N-terminal) pro-brain-type natriuretic peptide; TTE, transthoracic echocardiography. ^a^ Individual components of the Sartorelli score.

At the same time, limited availability, expertise, and procedural risks restrict the use of EMB and CMR in real-world practice (44, 45). Moreover, imperfect sensitivity leads to ambiguities: EMB is prone to sampling errors in patchy involvement (9, 30); CMR may yield false-negative results (31), e.g., due to moderate sensitivity of the Lake Louise Criteria for EM (32). Although CMR and/or EMB should clearly be pursued in suspected EM according to disease severity, as recently reiterated for myocarditis in general (4), EAD-associated cardiac dysfunction may occur despite inconclusive/unavailable EMB/CMR findings, and EM should therefore also be considered on the basis of complementary evidence, including biomarkers. To facilitate translation of our findings into clinical practice, we propose an author-developed algorithm for cardiac baseline assessment and risk stratification in EM (Figure 6).

Analyzing immunosuppressive treatment patterns poses a challenge due to the retrospective design and the lack of an EM-specific protocol during the study period. High-dose GC formed the backbone of induction therapy; however, more than half of patients remained on GC after a median of 34 months. Non-GC agents broadly followed common practice for management of the underlying EAD but varied substantially between patients (32). In EGPA, CYC and RTX were used in only 12/29 (41%, including one dEM) and 3/29 (10%), respectively; one patient received both drugs, leaving 15/29 EGPA patients without either agent during induction. One diagnosis predated the first European treatment recommendations, and in one case, escalation of immunosuppression was not pursued due to patient preference (23). For the remaining cases, the rationale for not adding CYC or RTX could not be reliably reconstructed from routine-care records. Since available guidance for severe EGPA does not stratify EM treatment by cardiac phenotype or severity, this heterogeneity likely reflects the general uncertainty surrounding treatment decisions in EM, evolving therapeutic options, and individualized real-world care.

After approval of mepolizumab (12/2015) and benralizumab (01/2018) in Germany, anti-IL-5 therapies were used more frequently, often in the context of concomitant eosinophilic asthma (21/29 EGPA including 3 dEM; 11/15 HES including 1 dEM). However, treatment was generally initiated during follow-up, as patients with acute cardiac disease had been excluded from pivotal clinical trials and EM-specific recommendations remain lacking (12).

HF pharmacotherapy was insufficient in approximately half of eligible patients, despite improving treatment rates and dosing over time. While polypharmacy-related adherence issues may contribute, underprescription appears to be the predominant barrier (35, 46). Documented HF therapy at baseline and follow-up was associated with numerically greater LVEF improvement, but this finding remains descriptive and cannot establish treatment effect. Three patients with positive baseline EMB/CMR improved substantially without HF therapy, suggesting an interplay between immunosuppression, spontaneous recovery, and patient-specific factors, particularly evidence of baseline inflammation.

Several limitations of this study need to be acknowledged. First, the quaternary referral setting was likely enriched for severe and complex cases, introducing selection bias. Local referral patterns, expertise, and individualized treatment decisions may limit generalizability to milder disease and other healthcare systems. In particular, access to specialized multidisciplinary care, advanced imaging, and biologic immunomodulatory drugs may vary substantially across settings and geographic regions with differing reimbursement pathways or resource availability (44, 47). Consequently, both the diagnostic work-up and treatment patterns observed in our cohort may not fully reflect routine practice elsewhere. A small proportion of EM may remain subclinical or minimally symptomatic (2). Because inclusion required the ESC criteria for clinically suspected myocarditis, such cases were likely underrepresented in the present cohort (19). Therefore, our findings primarily reflect clinically manifest EM and may overestimate disease severity at the population level.

Second, the retrospective single-center design limited control over data completeness, timing of assessments, and treatment allocation, thereby precluding causal inference. Treatment analyses were limited by heterogeneous follow-up intervals and the lack of systematic documentation of treatment continuity, relapses, and re-induction strategies. In particular, the 1FU assessment reflected the first available routine-care visit with cardiac evaluation after the induction phase rather than a predefined timepoint. Because the duration and composition of induction therapy varied substantially between patients, and follow-up intervals differed between the involved medical departments, 1FU cannot be interpreted as a standardized mid-term outcome assessment.

Composite scores were retrospectively derived from routine-care data and may have been affected by incomplete documentation, potentially introducing scoring imprecision. Compared with the r-FFS, which is predominantly based on routinely documented clinical variables, including major organ manifestations and general clinical parameters, the Sartorelli score relies on specialized cardiac investigations and is thus more susceptible to missing data.

The availability and timing of advanced diagnostics were not standardized and susceptible to indication bias, limiting assessment of their independent prognostic value. Furthermore, associations of CMR findings and cardiac status at fFU require cautious interpretation because they are cross-sectional. Prospective studies with serial CMR and standardized cardiac assessment are needed to determine whether progressive LV dilatation is associated with adverse clinical outcomes in EM.

Accordingly, associations between biomarkers, scores, imaging findings, treatment patterns, and outcomes should be interpreted exploratory and not as evidence of causality; external validation is required before they can inform routine care.

Owing to the rarity of EM, we included patients with different underlying EAD, reflecting its occurrence as a syndrome across etiologies in clinical reality. Although a recent registry of EMB-proven EM found no major differences in cardiac function or post-discharge events among EGPA, hypersensitivity, HES, and idiopathic EM (33), conclusions based on the combined EAD cohort warrant caution. Despite being substantial for such a rare condition, the cohort size of 51 patients limited statistical power, particularly for the smaller idiopathic EM and dEM subgroups, analyses with missing data or few events, as well as generalizability to more homogeneous populations such as EGPA-only cohorts. We therefore restricted formal subgroup comparisons to EGPA and i-HES and used parsimonious statistical models.

Not all cases were histologically confirmed by EMB, and diagnostic misclassification cannot be entirely excluded. However, throughout most of the study period, the 2013 ESC position statement (19) served as the relevant ESC diagnostic framework for clinically suspected myocarditis and bridged clinical and tissue-based diagnosis, whereas the 2025 ESC myocarditis guidelines were published only after the study period had ended (4).

These limitations define requirements for future research. Prospective multicenter studies should enroll patients across diverse referral settings, apply harmonized diagnostic and etiologic criteria, and use predefined follow-up windows. Standardized CMR acquisition with expert or central review, together with systematic recording of EMB findings where indicated, will be essential. Data collection should capture cardiac severity and treatment exposure—including induction regimens, GC dose and taper, rationale for immunomodulatory drug selection, relapses, re-induction, and concomitant HF therapy—to identify patients who may benefit from escalation beyond GC. Prespecified clinical, functional, imaging, and patient-centered endpoints are needed to validate the prognostic relevance of NT-proBNP, LVEF, and the Sartorelli score and to develop EM-specific biomarkers and clinically applicable risk-stratification approaches.

## Conclusion

EM affects multiple domains of cardiac function; however, diagnosis and risk-adapted treatment remain challenging. In this real-world cohort, baseline LVEF, NT-proBNP, and the Sartorelli score were associated with later cardiac function and LV remodeling, while baseline evidence of myocardial inflammation on CMR and/or EMB was associated with subsequent LVEF improvement. Together, our findings support the use of multimodal baseline biomarkers to identify patients at risk for persistent cardiac dysfunction, including those with reduced LVEF, markedly elevated NT-proBNP, or a high Sartorelli score at baseline. Such risk stratification could facilitate individualized management and improve the likelihood of cardiac recovery. The high glucocorticoid burden and heterogeneity in immunomodulatory and HF treatment underscore the need for novel therapeutic strategies in acute eosinophilic cardiomyopathy. Prospective multicenter studies should validate these findings, identify EM-specific biomarkers, and test whether biomarker-guided risk stratification improves outcomes.

## Data Availability

The original contributions presented in the study are included in the article/Supplementary material; further inquiries can be directed to the corresponding authors.
