# Supplementary material for: Heart failure and predictors of outcome in eosinophilic myocarditis: a real-world cohort study
Source: Front Med (Lausanne). 2026 Aug 26;13:1887831. doi: 10.3389/fmed.2026.1887831 (PMC13553266; doi:10.3389/fmed.2026.1887831)
Supplement: Supplementary file 1 [file Data_Sheet_1.docx]

**Supplementary Figures**

**Supplementary Figure 1. Course of laboratory parameters in EM patients illustrated by Tukey’s boxplots.** Unpaired data including all individuals. 1Dx=initial diagnosis; 1FU=first follow-up; fFU=final follow-up; n.d.=not determined; (NT-pro)BNP=(N-terminal pro)-brain-type natriuretic peptide; Box=interquartile range (IQR); horizontal line=median; whiskers=1.5×IQR; points=outliers; cross=mean.

*Alt text: Graph depicting the course of laboratory parameters from diagnosis to first follow-up, and final follow-up, illustrated with eight boxplots and individual data points. All individual values are included, regardless of whether a paired value at another timepoint is available.*

**Supplementary Figure 2. Association of left-ventricular volumetric indices with cardiac status at final follow-up. (A)** Last-available left-ventricular end-systolic/-diastolic volume index (LVESVI/LVEDVI) were related to log_10_-transformed N-terminal pro-brain-type natriuretic peptide (NT-proBNP) at final follow-up (fFU) using correlation and linear regression. **(B)** Associations with any cardiac symptoms and rhythm-related events at fFU are shown as odds ratios with 95% confidence intervals from multivariable logistic regression. Rhythm-related events were defined as documented palpitations and/or rhythm or conduction abnormalities recorded on 12-lead ECG, Holter ECG monitoring, or clinical documentation. LV=left-ventricular; straight line=simple linear regression line. **p<0.01; ***p<0.001

*Alt text: Two-panel figure showing exploratory associations of final CMR-derived LV volume indices with NT-proBNP and clinical outcomes. Panel A presents spearman correlation and linear regression of LVESVI and LVEDVI versus log_10_-transformed NT-proBNP at final follow-up. Panel B shows odds ratios with 95% confidence intervals for any cardiac symptoms and rhythm-related clinical events per 10 mL/m² increase in LVESVI or LVEDVI; larger LV volumes were associated with higher odds of both outcomes. Squares indicate left ventricular end-systolic and circles left ventricular end-diastolic volume index.*

**Supplementary Figure 3. Association of baseline laboratory parameters with cardiac function in EM patients.** 1Dx=initial diagnosis; AEC=absolute eosinophil counts; BSA=body surface area; CRP=C-reactive protein; Last=last available values; LVEF=Left-ventricular ejection fraction; LVESVI/LVEDVI=LV end-systolic/-diastolic volume indices; REC=relative eosinophil counts. AEC/REC were log_10_-transformed for statistical analysis; if values were 0, these were replaced by one tenth of the lowest non-zero value prior to log_10_-transformation. The unexpected association between higher peripheral eosinophil counts and better baseline LVEF may reflect eosinophilic tissue infiltration in acute disease, potentially driven by local myocardial interleukin-5 production, resulting in normal peripheral counts despite more pronounced LVEF impairment [1,21,33]. Accordingly, patients lacking eosinophilia at baseline had numerically lower LVEF than those with elevated AEC/REC (36±17% vs. 50±16%; p=0.052). Straight line=simple linear regression line; p-values refer to regression analysis; ns=not significant; *p<0.05.

*Alt text: Univariable linear regression plots in a multi-panel figure relating laboratory baseline biomarkers to left ventricular ejection fraction and indexed left ventricular volumes.*
