# Supplementary material for: Heart failure and predictors of outcome in eosinophilic myocarditis: a real-world cohort study
Source: Front Med (Lausanne). 2026 Aug 26;13:1887831. doi: 10.3389/fmed.2026.1887831 (PMC13553266; doi:10.3389/fmed.2026.1887831)
Supplement: Supplementary file 2 [file Data_Sheet_2.docx]

**Supplementary Tables**

**Supplementary Table 1.** 2013 European Society of Cardiology diagnostic criteria for clinically suspected myocarditis. Adapted from [19].

| **Clinical criteria** | **Diagnostic criteria** |
| --- | --- |
| 1. Acute chest pain, pericarditic, or pseudo-ischemic | 1. *Electrocardiogram/Holter/stress test features*: Newly abnormal 12 lead ECG and/or Holter and/or stress testing with any of the following: I to III degree atrioventricular block, or bundle branch block, ST/T wave change (ST elevation or non ST elevation, T wave inversion), sinus arrest, ventricular tachycardia or fibrillation and asystole, atrial fibrillation, reduced R wave height, intraventricular conduction delay (widened QRS complex), abnormal Q waves, low voltage, frequent premature beats, supraventricular tachycardia |
| 2. New-onset (days up to 3 months) or worsening of: dyspnea at rest or exercise, and/or fatigue, with or without left and/or right heart failure signs |  |
| 3. Subacute/chronic (> 3 months) or worsening of: dyspnea at rest or exercise, and/or fatigue, with or without left and/or right heart failure signs | 2. *Myocardiocytolysis markers:* Elevated Troponin T and/or Troponin I |
| 4. Palpitation, and/or unexplained arrhythmia symptoms and/or syncope, and/or aborted sudden cardiac death | 3. *Functional and structural abnormalities on cardiac imaging (echo/angio/CMR)*: New, otherwise unexplained LV and/or RV structure and function abnormality (incl. incidental finding in apparently asymptomatic subjects): regional wall motion or global systolic or diastolic function abnormality, with or without ventricular dilatation, with or without increased wall thickness, with or without pericardial effusion, with or without endocavitary thrombi |
| 5. Unexplained cardiogenic shock | 4. *Tissue characterization by CMR*: Edema and/or late-gadolinium enhancement of myocarditic pattern. |

*Patients were diagnosed with “clinically suspected myocarditis” if ≥1 clinical criterion and ≥1 diagnostic criterion was present. Angiographically detectable coronary artery disease and known pre-existing cardiovascular disease or other extra-cardiac causes that could explain the syndrome had to be excluded. If the patient was asymptomatic ≥2 diagnostic criteria had to be met.*

*CMR=cardiac magnetic resonance imaging; LV=left ventricular; RV=right ventricular.*

**Supplementary Table 2.** Components of clinical composite scores: Revised Five-Factor-Score (r-FFS) and Sartorelli score.

| **Scoring** | **r-FFS** [16] | **Scoring** | **Sartorelli** [6] |
| --- | --- | --- | --- |
| **+1** | Age > 65 years | **+1** | Rhythm and/or repolarization abnormalities on electrocardiogram |
| **+1** | Cardiac symptoms (e.g., pulmonary edema; excluding laboratory values and echocardiography) | **+2** | Increased serum troponin |
| **+1** | Gastrointestinal involvement | **+2** | Hypokinetic areas and/or left-ventricular dysfunction on transthoracic echocardiogram |
| **+1** | Renal insufficiency (creatinine >150 µmol/L) | **+1** | Myocardial edema and/or early- and/or late gadolinium enhancement on cardiac magnetic resonance imaging |
| **+1** | Absence of ear-nose-throat involvement |  |  |
| **Sum** | **5 points** | **Sum** | **6 points** |

**Supplementary Table 3.** Summary of European Society of Cardiology (ESC) class I recommendations for heart failure (HF) pharmacotherapy stratified by publication period.

| **Time period** | **ESC guideline** | **HF classification** | **Recommended drugs** |
| --- | --- | --- | --- |
| **2008**–**2011** | 2008 [26] | LVEF≤40% | **ACEi** (all patients)  **Betablockers** (all stable patients)  **Spironolactone** (severe symptoms and LVEF≤35% despite optimal treatment) |
| **2012**–**2015** | 2012 [27] | “Systolic HF” /  LVEF≤40% / “HF-REF” | **ACEi** (all patients) or **ARB** (if ACEi not tolerated)  **Betablocker** (all patients)  **MRA** (spironolactone/eplerenone) (severe symptoms and LVEF 35% despite optimal treatment) |
| **2016**–**2020** | 2016 [28] | „HFrEF“ (LVEF≤40%) | **ACEi** (all patients) or **ARB** (if ACEi not tolerated/contraindicated) or **ARNI** (replacing ACEi when still symptomatic and tolerating ACEi)  **Betablocker** (all patients)  **MRA** (spironolactone/eplerenone) (severe symptoms and LVEF 35% despite optimal treatment) |
| **2021**–**present** | 2021 [15] | „HFrEF“ (LVEF≤40%) | **ACEi** (all patients) or **ARB** (if ACEi not tolerated) or **ARNI** (replacing ACEi/ARB when still symptomatic)  **Betablocker** (all patients)  **MRA** (spironolactone/eplerenone) (all patients)  **SGLT2i** (all patients) |
| **2023**–**present** | 2023 [29] | „HFmrEF“ (LVEF 41–49%)  „HFpEF“(LVEF≥50%) | **SGLT2i** (all patients) |

Of patients with data on HF therapy, earliest diagnosis was made in April 2008, and latest follow-up was in May 2025. Therefore, ESC guidelines since 2008 were included in the analysis.

ACEi=angiotensin-converting enzyme inhibitors; ARB=angiotensin receptor blockers, ARNI=angiotensin receptor-neprilysin inhibitor (sacubitril/valsartan); HFrEF=HF with reduced ejection fraction; HFmrEF=HF with mildly reduced ejection fraction; HFpEF=HF with preserved ejection fraction; MRA=mineralocorticoid receptor antagonist, SGLT2i=sodium-glucose cotransporter-2 inhibitor.

**Supplementary Table 4.** Individual induction treatment strategies in patients with eosinophilic myocarditis (EM).

| **Patient ID (sex)** | **Diagnosis** | **Treatment** | **Patient ID (sex)** | **Diagnosis** | **Treatment** |
| --- | --- | --- | --- | --- | --- |
| 1 (male/m) | EGPA | GC (Pulse); CYC | 27 (f) | EGPA | GC (Pulse); CYC |
| 2 (female/f) | EGPA | GC, MEP | 28 (m) | i-HES | GC (Pulse) |
| 3 (f) | EGPA | GC | 29 (m) | EGPA | GC (Pulse); NSAID; COL |
| 4 (m) | i-HES | GC | 30 (m) | EGPA | GC |
| 5 (m) | dEM | NSAID | 31 (f) | EGPA | GC |
| 6 (f) | EGPA | GC | 32 (f) | EGPA | GC; CYC |
| 7 (m) | EGPA | GC (Pulse); NSAID | 33 (m) | i-HES | GC |
| 8 (f) | i-HES | GC (Pulse) | 34 (m) | EGPA/dEM | GC (Pulse); NSAID; COL |
| 9 (m) | EGPA/dEM | GC (Pulse); CYC; BEN | 35 (m) | i-HES | GC; MEP |
| 10 (f) | EGPA | GC | 36 (m) | i-HES | GC; CYC; MEP |
| 11 (f) | EGPA | GC; RTX | 37 (m) | idiopathic | GC (Pulse); NSAID |
| 12 (m) | EGPA | GC (Pulse); CYC; BEN; COL | 38 (f) | idiopathic | GC (Pulse) |
| 13 (m) | EGPA | GC (Pulse); CYC; BEN | 39 (m) | i-HES | GC (Pulse);MEP |
| 14 (f) | EGPA | GC (Pulse); CYC; RTX; IVIG; PLEX | 40 (f) | idiopathic | GC (Pulse); AZA; CYA |
| 15 (m) | dEM | GC | 41 (m) | EGPA | GC (Pulse); AZA |
| 16 (m) | EGPA | GC (Pulse) | 42 (f) | i-HES | GC; MEP |
| 17 (f) | i-HES | GC (Pulse) | 43 (f) | dEM | GC (Pulse); NSAID; COL |
| 18 (f) | EGPA | GC (Pulse); CYC; IVIG | 44 (f) | i-HES | GC (Pulse) |
| 19 (m) | EGPA | GC; CYC | 45 (f) | EGPA | GC |
| 20 (m) | i-HES | GC (Pulse); CYC; HU | 46 (f) | EGPA/dEM | n.d.^a^ |
| 21 (f) | i-HES/dEM | GC (Pulse); NSAID | 47 (f) | i-HES | GC; CYC |
| 22 (m) | EGPA | GC (Pulse); CYC | 48 (m) | EGPA | GC; CYC |
| 23 (f) | EGPA | GC; CYC; NSAID; COL | 49 (m) | EGPA | GC (Pulse); NSAID |
| 24 (f) | i-HES | GC (Pulse); CYC | 50 (f) | EGPA | / ^b^ |
| 25 (f) | i-HES | GC | 51 (f) | EGPA | GC (Pulse); RTX |
| 26 (m) | i-HES | GC |  |  |  |

*Patient-level data are presented using study-specific pseudonymized identifiers. Induction treatment refers to therapies initiated for the acute episode of EM or within the initial induction period. AZA=azathioprine; BEN=benralizumab; COL= colchicine; CYC=cyclophosphamide; dEM=drug-associated EM; EGPA=eosinophilic granulomatosis with polyangiitis; GC=glucocorticoids; i-HES=idiopathic hypereosinophilic syndrome; MEP=mepolizumab; NSAID=non-steroidal anti-inflammatory drug; HU=hydroxyurea, CYA=cyclosporin A; RTX=rituximab.*

*^a^ At timepoint of symptom onset (chest pain, dyspnea), patient 46 was initially misdiagnosed (Tako-tsubo cardiomyopathy) following a rule-out of CAD; elevated troponin and ECG abnormalities (T-inversions) normalized over time and cardiac magnetic resonance (CMR) in later disease course initially showed isolated edema without late-gadolinium enhancement (LGE), why she was only started on maintenance treatment for severe eosinophilic asthma and polyposis (MEP; dupilumab). Following peripheral eosinophilia, CMR showed unambiguous signs of acute myocarditis with new edema and LGE 11 months later, why remission induction with GC and CYC was initiated. EGPA/dEM was diagnosed.*

*^b^ Patient 50 was already on EGPA maintenance treatment with MEP and methotrexate when she presented with clinically suspected myocarditis including new troponinemia and CMR showing subendocardial LGE. Since the patient was oligosymptomatic and coronary angiography excluded CAD, endomyocardial biopsy (EMB) was delayed. When EMB was performed, it presented with ambiguous post-inflammatory/fibrotic findings, why shared decision was to continue MEP and methotrexate, rather than to initiate GC, CYC and/or RTX therapy.*

**Supplementary Table 5.** Therapy and outcome stratified to heart failure (HF) classification at baseline.

|  | **HFrEF, LVEF ≤40%**  **(n=16)** | | **HFmrEF, LVEF 41**–**49%**  **(n=7)** | | **HFpEF, LVEF ≥50%**  **(n=19)** | | **p** |
| --- | --- | --- | --- | --- | --- | --- | --- |
|  | value | n with data | value | n with  data | value | n with  data |  |
| **Therapy** | | | | | | | |
| **Glucocorticoids, mg/d** | 250 [80–1000] | 16 | 90 [64–325] | 6 | 150 [60–1000] | 18 | 0.32 |
| **Glucocorticoids, mg/kg/d** | 3.8 [1–12] | 16 | 1 [1–5] | 6 | 1.8 [1–10] | 18 | 0.49 |
| **Cyclophosphamide, n (%)** | 5 (31) | 16 | 4 (57) | 7 | 4 (21) | 19 | 0.24 |
| **Cyclophosphamide, mg (cum.**^b^**)** | 5915±860 | 16 | 7700±1371 | 7 | 6330±1749 | 19 | 0.16 |
| **Anti-IL-5-bDMARD, n (%)** | 12 (75) | 16 | 3 (43) | 7 | 11 (61) | 18 | 0.33 |
| **Outcome** | | | | | | | |
| **Death, n (%)** | 3 (19) | 16 | 2 (29) | 7 | 0 (0) | 19 | 0.053^a^ |
| **Remaining symptoms, n (%)** | 10 (63) |  | 4 (57) |  | 2 (11) |  | **0.002^a^** |
| **Symptom-free, n (%)** | 6 (37) |  | 3 (43) |  | 16 (89) |  | **0.002^a^** |

*Anti-IL-5-bDMARD=anti interleukin-5 biological disease-modifying antirheumatic drug (i.e. mepolizumab, benralizumab); HFrEF=heart failure with reduced ejection fraction; HFmrEF=HF with mildly reduced ejection fraction; HFpEF=HF with preserved ejection fraction; LVEF=left-ventricular ejection fraction.*

*^a^* *For statistical analysis of outcomes, HFrEF/HFmrEF were compared to HFpEF.*

*^b^ Refers to cumulative cyclophosphamide dose after completion of induction.*
